# Supplementary material for: Mining of QTLs for Spring Bread Wheat Spike Productivity by Comparing Spring Wheat Cultivars Released in Different Decades of the Last Century
Source: Plants (Basel). 2024 Apr 12;13(8):1081. doi: 10.3390/plants13081081 (PMC11055096; doi:10.3390/plants13081081)
Supplement: Supplementary file 1 [file plants-13-01081-s001.zip › plants-2904057 File S1.pdf]

File S1. Manhattan plots and QQ-plots for each of the three years of field study: 2021 - 2023

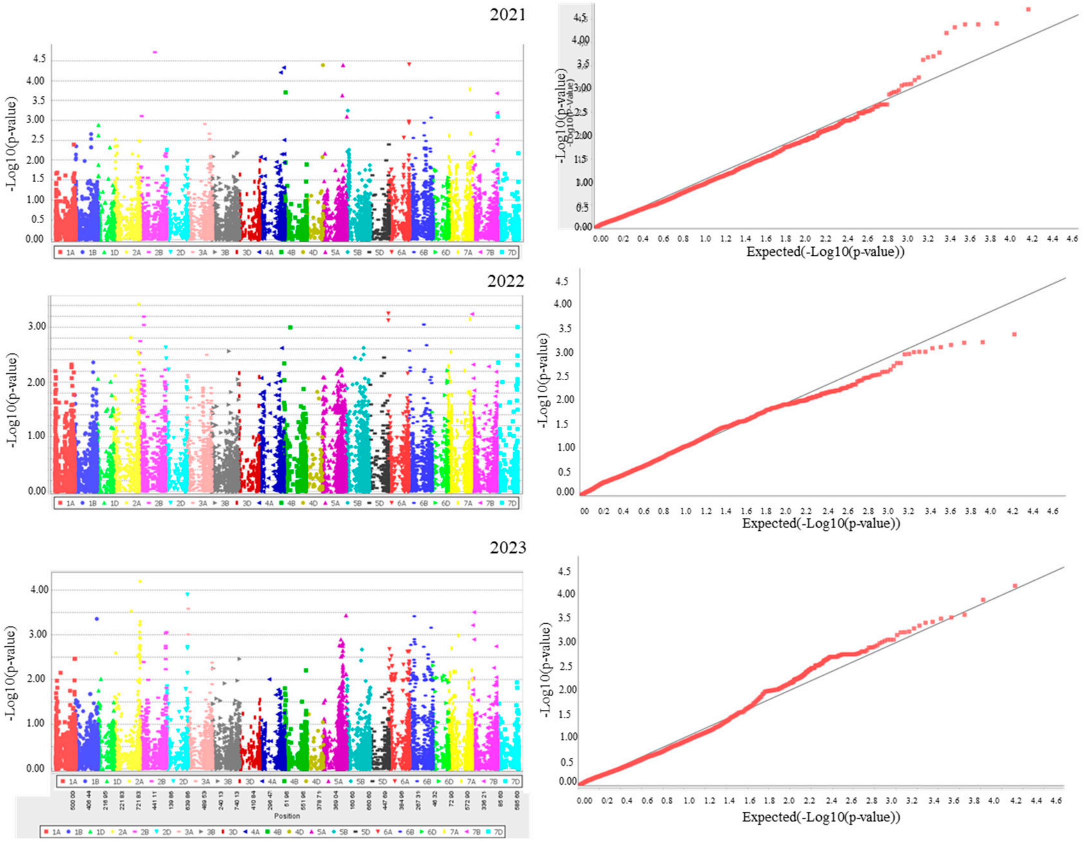

(a)

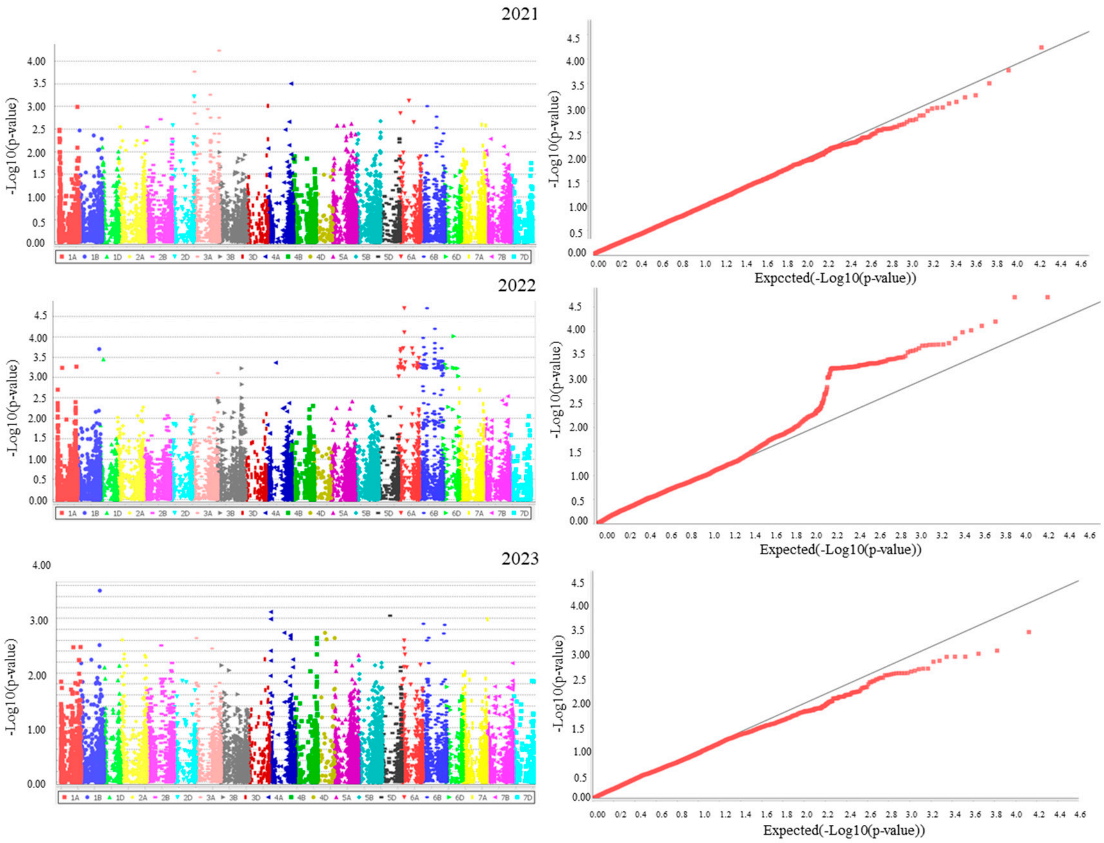

(b)

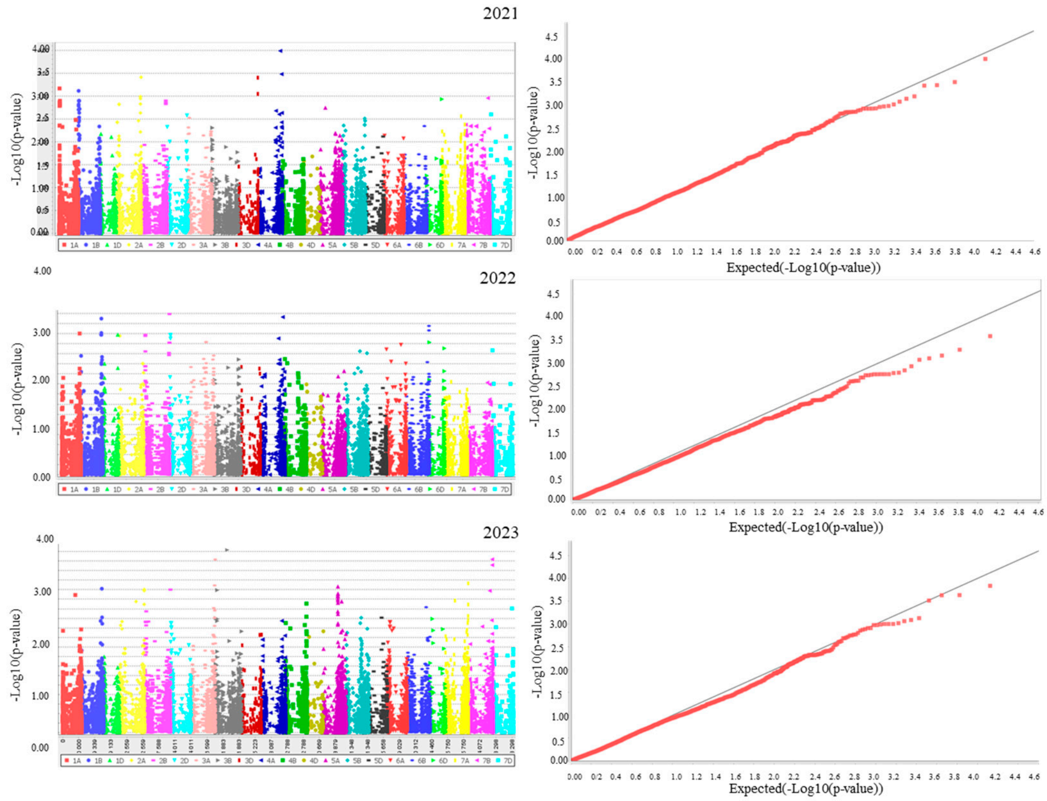

(c)

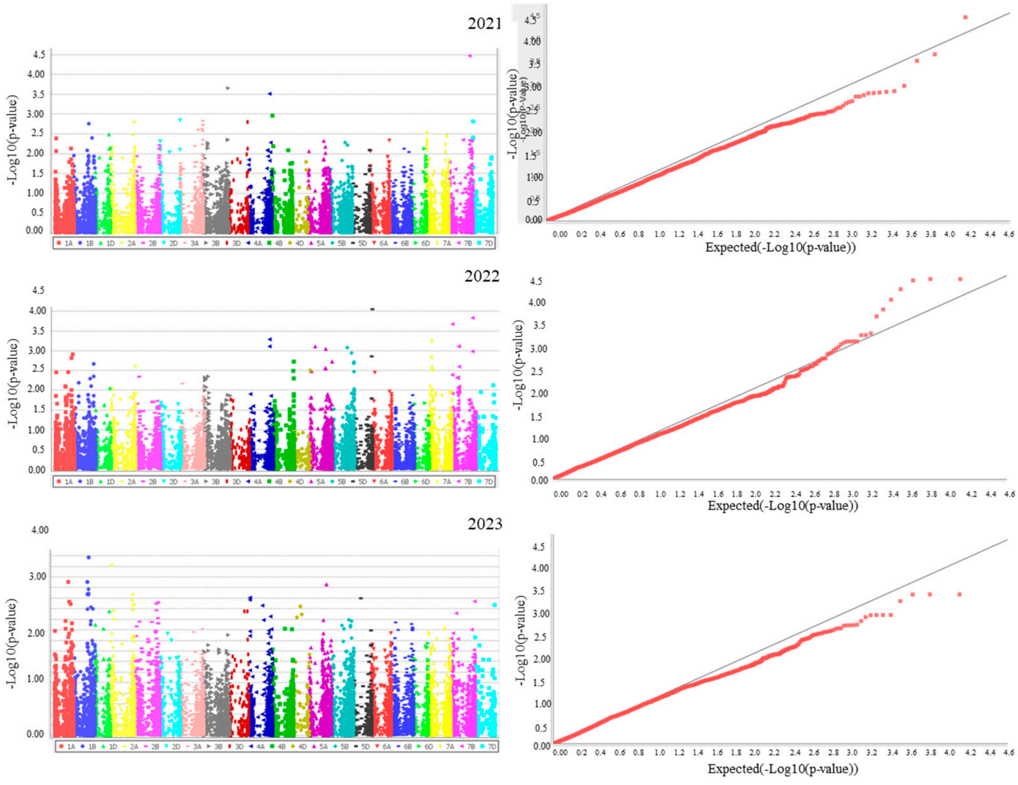

(d)

Figure S1. Manhattan plots and QQ-plots of 4 agronomic characters: (a) RL; (b) SL; (c) TGW; (d) SN. The red dots and gray straight lines on the QQ-plots indicate the experimental value and the expected value, respectively
