# Supplementary material for: Mining of QTLs for Spring Bread Wheat Spike Productivity by Comparing Spring Wheat Cultivars Released in Different Decades of the Last Century
Source: Plants (Basel). 2024 Apr 12;13(8):1081. doi: 10.3390/plants13081081 (PMC11055096; doi:10.3390/plants13081081)
Supplement: Supplementary file 1 [file plants-13-01081-s001.zip › plants-2904057 File S2.pdf]

File S2.

Table S1. Test weight values for the most contrast cultivars

VIR/IPK catalog No.	Cultivar	Origin. Accession status	Index, g/L
Cultivars with high values			
632855	Saksonka	Russia. Landraces	830
632897	Novosibirskaya-89	Russia. Modern improved cultivars	829
632898	Novosolyanskaya	Russia. Cultivars released in 1951–1991	832
632980/TRI 3461	Sommergrannen	Germany. Landraces	823
Cultivars with low values			
632920	Eritrospermum-28	Russia. Cultivars released in 1951–1991	622
632922/TRI 561	HeinesJaphet	Germany. Cultivars released before 1950	648
632923/TRI 562	Hohenheimer 25	Germany. Cultivars released before 1950	646
632925/TRI 766	StrubesWeissaehrigerFruehe	Germany. Cultivars released before 1950	635
632948/TRI 811	Erbachshofer	Germany. Cultivars released before 1950	647
632949/TRI 813	v. RuemkersSommer-Dickkopf	Germany. Cultivars released before 1950	638

Table S2. Grain protein content values for the most contrast cultivars

VIR/IPK catalog No.	Cultivar	Origin. Accession status	Index, %
Cultivars with high values			
632852	Noe-zapadnosibirskii	Russia. Landraces	20.4
632864	Tarskaya-2	Russia. Cultivars released in 1951–1991	18.7
632879	Amurskaya-90	Russia. Modern improved cultivars	18.4
632920	Eritrospermum-28	Russia. Cultivars released in 1951–1991	20.7
632921/TRI 559	Peragis	Germany. Cultivars released before 1950	18.1
632922/TRI 561	HeinesJaphet	Germany. Cultivars released before 1950	19.1
632923/TRI 562	Hohenheimer 25	Germany. Cultivars released before 1950	18.8

Cultivars with low values			
632828	Akmolinka	Russia. Landraces	12.3
632829	Alenkaya-Uymonskaya	Russia. Landraces	12.8
632831	Albidum-3700	Russia. Cultivars released before 1950	12.2
632840	Duvanka-501	Russia. Cultivars released before 1950	12.8
632843	Krasnoyarskaya-1103	Russia. Cultivars released in 1951–1991	12.3
632899	Omskaya-11	Russia. Cultivars released in 1951–1991	12.8
632900	Omskaya-9	Russia. Cultivars released in 1951–1991	12.6
632902	Pyrothrix-28	Russia. Cultivars released in 1951–1991	12.5
632903	Priobskaya	Russia. Cultivars released in 1951–1991	12.4
632904	Rodina	Russia. Cultivars released in 1951–1991	11.5
632907	Sibakovskaya-3	Russia. Cultivars released in 1951–1991	12.7
632909	Sibiryachka-4	Russia. Cultivars released in 1951–1991	12.5
632910	Sibiryachka-8	Russia. Cultivars released in 1951–1991	12.8
632978/TRI 3449	EndressFichtelgebirgs	Germany. Cultivars released before 1950	12.9
632981/TRI 3664	Sommergrannen	Germany. Cultivars released in 1951–1991	12.9

Table S3. Sedimentation values for the most contrast cultivars

VIR/IPK catalog No.	Cultivar	Origin. Accession status	Index, mL
Cultivars with high values			
632871	Tsezium-31	Russia. Cultivars released before 1950	80
632876	Altayskaya 60	Russia. Modern improved cultivars	80
632887	Irtyshanka-10	Russia. Cultivars released in 1951–1991	81
632949/TRI 813	v. RuemkersSommer-Dickkopf	Germany. Cultivars released before 1950	80
632995/TRI 8146	Probat	Germany. Cultivars released in 1951–1991	80
633006/TRI 9529	Kleiber	Germany. Cultivars released in 1951–1991	82
Cultivars with low values			
633007/TRI 9731	Carola	Germany. Cultivars released in 1951–1991	14
633009/TRI 10975	Bali	Germany. Cultivars released in 1951–1991	16
633010/TRI 10976	St.Samos	Germany. Cultivars released in 1951–1991	16

Table S4. Grain ash content values for the most contrast cultivars

VIR/IPK catalog No.	Cultivar	Origin. Accession status	Index, %

		Cultivars with high values	
632855	Saksonka	Russia. Landraces	1.68
632874	Selenga	Russia. Cultivars released in 1951–1991	1.68
632884	Grekum-114	Russia. Cultivars released in 1951–1991	1.53
632888	Kantegirskaya-89	Russia. Modern improved cultivars	1.68
632901	Orenburgskaya-7	Russia. Cultivars released in 1951–1991	1.67
632915	Fora	Russia. Modern improved cultivars	1.64
632938/TRI 798	BergersGiersdorfer	Germany. Cultivars released before 1950	1.69
632943/TRI 805	RaeckesWeisspelziger	Germany. Cultivars released before 1950	1.65
		Cultivars with low values	
632920	Eritrospermum-28	Russia. Cultivars released in 1951–1991	2.80
632922/TRI 561	HeinesJaphet	Germany. Cultivars released before 1950	2.64
633008/TRI 10174	Solo	Russia. Cultivars released in 1951–1991	2.64

Table S5. Flour color values for the most contrast cultivars

VIR/IPK catalog No.	Cultivar	Origin. Accession status	Index, %
		Cultivars with high values	
632831	Albidum-3700	Russia. Cultivars released before 1950	84.1
632874	Selenga	Russia. Cultivars released in 1951–1991	83.2
632880	Angara-86	Russia. Modern improved cultivars	83.1
632884	Grekum-114	Russia. Cultivars released in 1951–1991	83.0
632888	Kantegirskaya-89	Russia. Modern improved cultivars	83.7
632983/TRI 4931	HeinesFasan	Germany. Cultivars released in 1951–1991	83.1
		Cultivars with low values	
632840	Duvanka-501	Russia. Cultivars released before 1950	75.0
632848	Miltrum 553	Russia. Cultivars released before 1950	76.6
632922/TRI 561	HeinesJaphet	Germany. Cultivars released before 1950	74.8
